# RNA-editing in Basidiomycota, revisited

**DOI:** 10.1038/s43705-021-00037-9

**Published:** 2021-12-01

**Authors:** Byoungnam Min, Baojun Wu, Jill Gaskell, Jiwei Zhang, Christina Toapanta, Steven Ahrendt, Robert A. Blanchette, Emma Master, Daniel Cullen, David S. Hibbett, Igor V. Grigoriev

**Affiliations:** 1 US Department of Energy Joint Genome Institute, Lawrence Berkeley National Laboratory, Berkeley, CA USA; 2 Department of Plant and Microbial Biology, University of California, Berkeley, CA USA; 3 Biology Department, Clark University, Worcester, MA USA; 4 Statistics and Bioinformatics Group, School of Fundamental Sciences, Massey University, Palmerston North, New Zealand; 5 USDA Forest Products Laboratory, Madison, WI USA; 6 Department of Bioproducts and Biosystems Engineering, University of Minnesota, St. Paul, MN USA; 7 Department of Plant Pathology, University of Minnesota, St. Paul, MN USA; 8 Department of Chemical Engineering and Applied Chemistry, University of Toronto, Toronto, ON Canada

**Keywords:** Sequencing, Genomics

In fungi, adenosine-to-inosine (A-to-I) RNA-editing was shown to occur during sexual development and fruiting body formation in filamentous ascomycetes *Fusarium graminearum*, *F. verticillioides*, *Neurospora crassa*, *Sordaria macrospora*, and *Pyronema confluens* (Pezizomycotina) [1, 2], but absent during meiosis in the fission yeast *Schizosaccharomyces pombe* (Taphrinomycotina) [2]. In Basidiomycota, the first RNA-editing report was from *Ganoderma lucidum*, a mushroom-forming species of Polyporales, also during fruiting body formation [3]. Afterwards, Wu et al. reported RNA-editing events in vegetative mycelia of five other species of Polyporales [4, 5]. It was noted that the editing patterns of both *G. lucidum* and the five Polyporales fungi were not only different from the typical A-to-I editing but also showed that transitions (A- > G, G- > A, C- > U, U- > C) were preferred over transversions, leading mainly to synonymous changes in contrast to missense changes found in filamentous ascomycetes [3–5].

Wu et al. predicted RNA-editing sites by aligning transcriptomic reads to the reference genome assembly. However, that approach assumes that the reference assembly is complete, which is rarely the case. The majority of available fungal genome assemblies contain gaps and errors. Even in haploid genomes, multiple nearly-identical DNA regions, for example, transposons or tandem or segmental duplications, can be erroneously collapsed into single fragments, or misassembled. Therefore, RNA reads mapped to such collapsed regions would correspond to distinct genomic regions with 100% coverage and identity and thus would not represent transcripts of the same genes. To avoid this issue, gDNA reads can be aligned and analyzed simultaneously with RNA reads. When a potential RNA-editing site has the same single nucleotide variants as in the gDNA reads alignment, the site should be excluded from candidate RNA-editing sites.

We used this approach to reassess RNA-editing previously reported by Wu et al. for five species of Polyporales [4, 5]. When we compared the polymorphisms derived from the alignment of gDNA reads with those derived from RNA reads, a substantial fraction of these RNA and DNA polymorphisms did match in both the positions and nucleotide substitution types (Fig. 1). From the previously reported 184–1761 RNA-editing sites predicted based on the transcriptome-only analysis [4, 5], 148–1690 (60.68–98.31%) showed matching RNA and DNA polymorphisms and were removed (Table 1). For *Antrodia sinuosa*, DNA and RNA were extracted from different strains [4], which explains more remaining sites (568–923) than in the other four species (8–71).

**Fig. 1:**
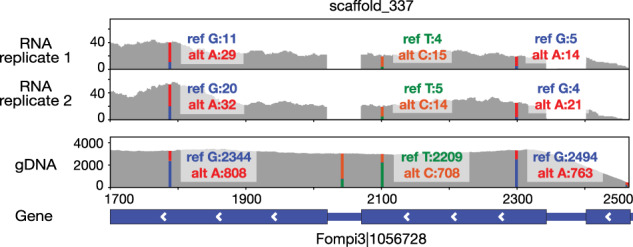
Previously reported putative RNA-editing sites that show the identical type of polymorphism in the gDNA reads alignment. The RNA samples were extracted from *Fomitopsis pinicola* grown in spruce for ten days.

**Table 1:** Previously reported RNA-editing in the five Polyporales fungi.

Species	Sample^a^	Previously reported RNA editing sites	Matching variants in gDNA read alignments^b^	Absent in the new RNA BAMs^c^	Matching variant in gDNA reads^d^	Different RNA and DNA sources
*Daedalea quercina* L-15889	Aspen-5D	435	421	13	0	1
	Pine-5D	355	347	8	0	0
	Spruce-5D	365	356	8	0	1
*Fomitopsis pinicola* FP-58527 SS1	Aspen-5D	1568	1510	45	13	0
	Pine-5D	1761	1690	53	18	0
	Pine-10D	753	716	27	9	1
	Pine-30D	1067	1022	34	11	0
	Spruce-5D	1288	1244	32	12	0
	Spruce-10D	901	863	33	5	0
	Spruce-30D	1098	1051	38	9	0
*Laetiporus sulphureus* 93-53	Aspen-5D	1328	1297	26	5	0
	Pine-5D	1339	1314	21	4	0
	Spruce-5D	1304	1282	19	3	0
*Wolfiporia cocos* MD-104 SS10	Aspen-5D	184	148	35	0	1
	Pine-5D	212	173	38	1	0
	Spruce-5D	195	153	42	0	0
*Antrodia sinuosa*^e^	Aspen-5D	1141	698	139	-	304
	Pine-5D	1504	923	229	-	352
	Spruce-5D	936	568	110	-	258

^a^Sample nucleic acids were derived from woody substrates after 5, 10 or 30 days incubation [4, 5].

^b^The number of removed sites where gDNA read alignments show the same single-nucleotide variation.

^c^The number of sites absent in the alignments performed with the latest version of HISAT (v0.1.4-beta used for previous study and v2.1.0 used for this study).

^d^The number of removed sites where polymorphic RNA reads match DNA reads despite lack of alignments into the positions.

^e^Genomic DNA was extracted from LB1 strain while RNA was from LD5-1 strain.

Some of these remaining candidate sites did not show the single-nucleotide variations when the later version of HISAT alignment was used with intron length restriction (2000 bp). That is because the reads having supported the variations were decoupled and mapped to different regions or were not mapped due to the intron length limitation. In addition, when we searched reads pooled rather than aligned reads, we observed distinct groups of gDNA reads matching the polymorphic RNA reads with 100% identify of the entire length and thus corresponding to different parts of the genome (Table 1). After this multi-step filtering, all but four sites across four species were eliminated. The RNA polymorphism in the remaining four sites can be explained by the differences in isolates used for genome sequencing and for the transcriptomics studies by Wu et al. [4, 5] (Table 2). These polymorphisms were not confirmed in RNA reads used for genome annotation and obtained from the same isolates as DNA. Thus, there is no evidence of RNA editing during the vegetative growth in these five species of Polyporales.

**Table 2:** Genome and transcriptome data used in this study.

Species	Genome assembly	Genomic reads	Transcriptomes
*Antrodia sinuosa*^a^	mycocosm.jgi.doe.gov/Antsi1	SRP025451	SRP145284–145291
*Fomitopsis pinicola* FP-58527 SS1	mycocosm.jgi.doe.gov/Fompi3	SRP004032	SRP140951–140968
*Daedalea quercina* L-15889	mycocosm.jgi.doe.gov/Daequ1	SRP024551	SRP145276–145283
*Laetiporus sulphureus* 93-53	mycocosm.jgi.doe.gov/Laesu1	SRP025501	SRP164792–164802
*Wolfiporia cocos* MD-104 SS10	mycocosm.jgi.doe.gov/Wolco1	SRP002992	SRP145298–145306
*Coprinopsis cinerea* AmutBmut pab1-1	mycocosm.jgi.doe.gov/Copci_AmutBmut1	SRP053467	SRP179762

^a^Genomic DNA was extracted from LB1 strain while RNA was from LD5-1 strain.

We performed further analyses on how assembly methods can affect detecting RNA/DNA polymorphisms. We reassembled the *Daedalea quercina* genome that previously had been assembled by AllPathsLG [6]. The new assembly was built by SPAdes and resulted in more scaffolds (606 vs. 357), fewer contigs (924 vs. 1025), and slightly smaller assembly size (31.40 vs. 32.74 Mbp). We identified 37 (aspen), 47 (pine), and 45 (spruce) RNA polymorphism sites in the original assembly that correspond to pairs of separate regions in the new assembly, thus eliminating both DNA and RNA polymorphism at those sites. Nevertheless, the new assembly introduced 605 (aspen), 694 (pine), and 704 (spruce) new single nucleotide variants of RNA not found in the old assembly. Thus, the assemblers produce different misassemblies and can potentially suggest new false RNA polymorphisms.

Since the RNA of all five Polyporales species was extracted from mycelium, we extended our analysis to the homokaryon of *Coprinopsis cinerea* (Agaricomycetes, Agaricales) for which transcriptomic data were collected across several developmental stages as reported previously [7]. The samples included vegetative mycelium, secondary hyphal knot, stage 1 primordium, stage 2 primordium, young fruiting body (cap, lamellae, and stipe), and fruiting body (cap + lamellae and stipe). We used both genomic DNA and RNA reads to filter out the false RNA-editing sites derived from potential genome mis-assemblies. As a result, we obtained only two to six RNA variant sites after filtering which can be explained by DNA and RNA extracted from different isolates sequenced in independent studies [7, 8]. Thus, we found no evidence for RNA-editing events in *C. cinerea* during sexual development.

Similar transitions-over-transversions nucleotide patterns of RNA-editing were reported in *Ganoderma lucidum* [3]. The report suggested 8906 RNA-editing sites in *G. lucidum* using analysis of both gDNA and RNA reads, with 94 sites verified with Sanger sequencing [3]. While the reference genome was built by the one of two mating strains (monokaryon), the fruiting-body from which gDNA and RNA were extracted was formed by a dikaryon where two mating strains exist. *G. lucidum* is a heterokaryotic species, making the analysis more challenging because of the ploidy combined with segmental duplications. In order to confirm RNA editing in this species, two haplotypes need to be separated and analyzed independently with proper DNA and RNA reads mapped to each of them to identify haplotype-specific polymorphisms indicating possible mis-assemblies that need to be filtered out in analysis of RNA polymorphisms. This may be quite challenging, but the editing pattern reported in *G. lucidum* was similar to those we corrected in the haploid genomes of Polyporales in that transitions dominate transversions leading to mostly synonymous amino acid changes.

To conclude, we revisited the previously reported RNA-editing in five basidiomycetes, and extended analysis to one more species with transcriptionally profiled developmental stages. The previously reported sites of RNA-editing were artifacts caused by genome misassemblies or variations in the genomes of different isolates from which DNA and RNA were extracted. Furthermore, we suggest reevaluating the RNA-editing in the dikaryotic *G. lucidum* by separate analysis of its two haplotypes. We were also unable to detect RNA-editing patterns in any transcript profiles during development of *C. cinerea*, including sexual development. In short, after reevaluation of available genomes and transcriptomes we did not find evidence of RNA-editing in Basidiomycota.

## Methods and materials

We downloaded genomic and transcriptomic read data used in this study from the NCBI database (Table 2). The genome and transcriptome reads were de-duplicated to remove polymerase chain reaction products using Dedupe in BBTools (https://sourceforge.net/projects/bbmap/). Subsequently, the reads were quality controlled using TrimGalore (https://www.bioinformatics.babraham.ac.uk/projects/trim_galore/) by filtering out the reads with bad base quality (<20) and short length (<40). We aligned filtered genomic and transcriptome reads against the corresponding genome assemblies using Bowtie v2.3.5.1 (http://bowtie-bio.sourceforge.net) and HISAT v2.1.0 (http://www.ccb.jhu.edu/software/hisat/), respectively. The candidate RNA-editing sites were detected using JACUSA v1.3.0 [9] (call-2 --filter-flags 1024 --min-mapq1 0 --min-mapq2 0 --pileup-filter S). The variants supported with <20 total mapped reads, <5 or <10% of variant reads, and any sites with matching gDNA variants (>5% variants of mapped reads) were ignored. We used SPAdes v3.13.0 (http://cab.spbu.ru/software/spades) (--careful --cov-cutoff auto) to reassemble *D. quercina* genomic reads. The original and newly assembled genomes were aligned using NUCmer 4.0.0beta2 (http://mummer.sourceforge.net) to find corresponding sites. The RNA reads from the same isolates that DNA reads were generated were downloaded from MycoCosm (https://mycocosm.jgi.doe.gov/) [10].
